# Rapid nanomolding of nanotopography on flexible substrates to control muscle cell growth with enhanced maturation

**DOI:** 10.1038/s41378-021-00316-4

**Published:** 2021-11-05

**Authors:** Cong Wu, Chriss S. M. Chin, Qingyun Huang, Ho-Yin Chan, Xinge Yu, Vellaisamy A. L. Roy, Wen J. Li

**Affiliations:** 1 Department of Mechanical Engineering, City University of Hong Kong, Hong Kong, China; 2 Department of Biomedical Engineering, City University of Hong Kong, Hong Kong, China; 3 James Watt School of Engineering, University of Glasgow, Glasgow, G12 8QQ UK

**Keywords:** Nanofabrication and nanopatterning, Nanostructures

## Abstract

In vivo, multiple biophysical cues provided by highly ordered connective tissues of the extracellular matrix regulate skeletal muscle cells to align in parallel with one another. However, in routine in vitro cell culture environments, these key factors are often missing, which leads to changes in cell behavior. Here, we present a simple strategy for using optical media discs with nanogrooves and other polymer-based substrates nanomolded from the discs to directly culture muscle cells to study their response to the effect of biophysical cues such as nanotopography and substrate stiffness. We extend the range of study of biophysical cues for myoblasts by showing that they can sense ripple sizes as small as a 100 nm width and a 20 nm depth for myotube alignment, which has not been reported previously. The results revealed that nanotopography and substrate stiffness regulated myoblast proliferation and morphology independently, with nanotopographical cues showing a higher effect. These biophysical cues also worked synergistically, and their individual effects on cells were additive; i.e., by comparing cells grown on different polymer-based substrates (with and without nanogrooves), the cell proliferation rate could be reduced by as much as ~29%, and the elongation rate could be increased as much as ~116%. Moreover, during myogenesis, muscle cells actively responded to nanotopography and consistently showed increases in fusion and maturation indices of ~28% and ~21%, respectively. Finally, under electrical stimulation, the contraction amplitude of well-aligned myotubes was found to be almost 3 times greater than that for the cells on a smooth surface, regardless of the substrate stiffness.

## Introduction

Nanotopography refers to the specific features on surfaces generated at the nanoscale^1–3^. In nature, a wide variety of nanotopographies have been identified as functional surfaces, such as in lotus leaves and cicada wings^4–6^. The epidermis of a lotus plant has a hierarchically well-defined architecture, incorporating nano and microroughness, which remarkably reduces the adhesion force between water droplets and leaf surfaces, resulting in ultrahydrophobicity and self-cleaning properties. However the surface of the wings of cicada species has been demonstrated to possess bacteriostatic and bactericidal effects, which are attributed to nanoscale denticle structures with different densities and diameters on the cicada wings. Considering the tissues in a multicellular organism, nanotopography has also been found everywhere in the extracellular matrix (ECM) around cells^7–9^, which mainly consists of various fibrous proteins and proteoglycans^10^. For instance, parallelly aligned collagen fibrils approximately 50–200 nm in diameter make up the ECMs of the tendon and myocardium^11,12^, while the basement membranes of epithelia exhibit a complex texture with pits, bumps, grooves, and ridges in the nanometer range^13,14^. At the same time, natural ECMs, which provide multiple cues, such as surface topography and matrix stiffness, have also been observed to considerably regulate the growth and differentiation of embedded cells^15^. For example, surrounded by a highly ordered network of fibrous connective tissues, which consist of collagen fibers and display nanoscale topographical features, individual muscle fibers line up parallel to each other to form skeletal muscles^16–18^.

Despite the complexity of cellular microenvironments in vivo, cells are usually grown on flat and rigid substrates by using conventional in vitro cell culture techniques. As a result, skeletal muscle cells appear in a random arrangement in a laboratory dish, rather than their native organization of highly parallel alignment, potentially affecting many of their physiological properties^19^. Thus, a great deal of effort has been devoted to exploring the influences of biophysical cues on cell behaviors by mimicking the characteristics of natural ECMs^20–25^, and consequently, nanotopography has been identified as a key regulator to enable cell maturation and alignment^26–29^. Previous studies have demonstrated that grooves with widths ranging from 200 to 900 nm and depths ranging from 100 to 600 nm as well as fibers with diameters ranging from 200 to 760 nm are able to modulate cell growth, including adhesion, proliferation, and differentiation, and induce the parallel alignment of muscle cells^30–36^. Additionally, some research has further indicated that the critical dimension of nanoripple patterns that myoblasts can perceive and align along is 430 nm width by 100 nm depth, while the cells are randomly oriented on the surfaces with smaller patterns (i.e., width of 200–340 nm and depth of 40–80 nm)^32,33^. In addition, the groove depth has been reported to be more influential on cellular morphology and alignment than groove width^33^. In addition to nanotopography, other ECM cues, such as matrix stiffness, mechanical force, and electrical and chemical stimuli, have been investigated^37–39^. However, their synergistic effects on muscle cells have not been fully revealed in a quantitative manner.

Current technologies for the fabrication of ultrafine surface patterns at the nanoscale mainly include electron beam lithography, focused ion beam lithography, nanoimprint lithography, and various microscope-assisted nanolithography techniques—all these technologies exhibit high controllability and precision^40–45^. However, their application to biological studies is hindered by the use of harsh solvents, which may be harmful to the survival of cells. Moreover, mass production of surfaces with nanoscale patterns is currently also limited owing to the complicated, costly, low-efficiency, and time-consuming manufacturing processes of these technologies^46^. Consequently, fabricating substrates with nanotopographies using these methods is difficult to integrate into routine culturing of cells. Fortunately, commercially available optical discs with different recording modes, including *read-only*, *recordable*, and *rewritable* modes, already incorporate polycarbonate (PC) substrates patterned with an abundance of concentric ring-shaped nanogrooves, which can be used for cell culturing. Although these three types of optical discs have a round appearance in common, their interior layer structures are different. Compared with *read-only* discs, where the stored data are molded as pits and lands forming discontinuous nanogrooves, *rewritable* discs have a phase-transitioning metal alloy film (which is difficult to remove) covering the nanogrooved base surface. On the other hand, *recordable* discs (which have continuous nanogrooves) are the most convenient for separating optically transparent PC substrates from other structural layers; i.e., nanogrooves on substrates can be easily obtained through a fully biocompatible process. Therefore, *recordable* discs can be used directly to culture cells after proper preprocessing of the discs. These discs can also be used to rapidly manufacture polymer-based substrates with large-scale nanogrooves of various sizes using nanomolding techniques.

**Fig. 1:**
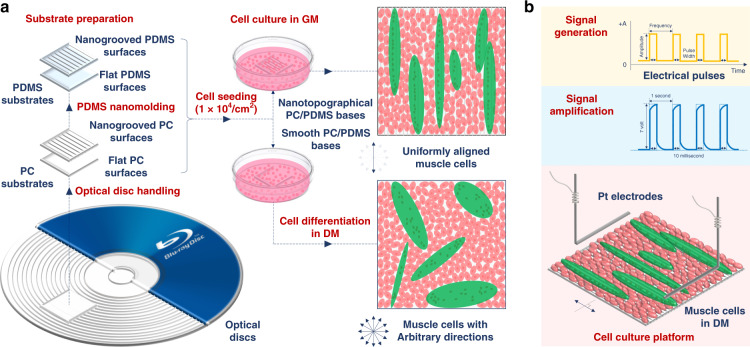
Experimental design of nanotopography-induced muscle cell growth. **a** Illustration of the experimental flow, including preparation of optical disc-based PC substrates, fabrication of PDMS substrates with/without nanoscale surface patterns through nanomolding, cell culture in growth medium for 3 days until overgrowth, and cell differentiation in differentiation medium for another 1 week. **b** Schematic of electric pulse stimulation on well-aligned myotubes

In the present research, CD-R, DVD-R, and BD-R (Blu-ray disc recordable) optical discs and their softer polydimethylsiloxane (PDMS) replicas were developed as cell culturing platforms. Although a variety of optical media, such as compact discs, phonograph records, and a series of diffraction gratings, have been previously used either directly or as templates for fabricating substrates for cell growth^28,47–49^, this work is the first to demonstrate that BD-R with a groove width of 100 nm and a groove depth of 20 nm can be applied to culture skeletal myoblasts. Based on this technique, a comprehensive investigation of the nanotopography-regulated growth of muscle cells was carried out, as shown in Fig. 1a, and the corresponding cellular behaviors, including adhesion, elongation, proliferation, differentiation, and maturation, were quantified in detail. Moreover, electrical stimulation was also conducted on myotubes cultured on substrates with different topographies to quantitatively compare the difference in cell contraction, as shown in Fig. 1b. This study provides new insights into the specific interactions between biophysical cues and muscle cell growth, extending the range of minimum groove sizes to which myoblasts can respond. In addition, this work also demonstrates that flexible substrates (e.g., PDMS) with nanotopographies made using optical discs as molds are suitable for routine cell culturing; i.e., the efficient and biocompatible nanotopography fabrication process presented here has great potential for application in muscle regeneration and transplantation research fields.

## Results

### Characterization of nanotopographical patterns

**Fig. 2:**
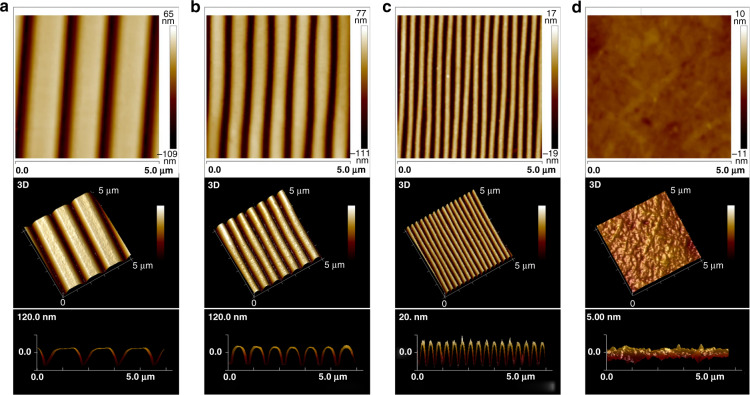
AFM images of nanotopographical and flat PC surfaces obtained from different optical media in 2D and 3D (top view and side view). **a** CD-R (groove width: 500 nm; ridge width: 900 nm; ridge height: 120 nm). **b** DVD-R (groove width: 300 nm; ridge width: 450 nm; ridge height: 120 nm). **c** BD-R (groove width: 100 nm; ridge width: 200 nm; ridge height: 20 nm). **d** Flat PC (average R_a_: 1.56 nm)

The patterned PC bases obtained from CD-R, DVD-R, and BD-R optical discs and flat PC substrates were measured via AFM and SEM, as shown in Fig. 2 and Fig. S1. From the results, the PC substrates from all three types of optical discs provided the nanotopographies of a repeating parallel groove/ridge with different dimensions of width and depth on their surfaces, while no pattern was found on the flat PC surface. In detail, the widths of the grooves were approximately 500, 300, and 100 nm, and the corresponding widths of the ridges were approximately 900, 450, and 200 nm for the CD-R, DVD-R, and BD-R discs, respectively. In addition, the grooved patterns on the CD-R and DVD-R substrates exhibited an identical depth of 120 nm, whereas the depth for BD-R was 20 nm. Thus, the dimensions of these surface nanostructures spanned the entire nanoscale range. More notably, the width and depth of the nanogrooves on the BD-R were less than the minimum ripple size that myoblasts could sense for alignment, as mentioned in previous literature^31^, which facilitated comprehensive research on nanotopography-induced muscle cell behaviors.

**Fig. 3:**
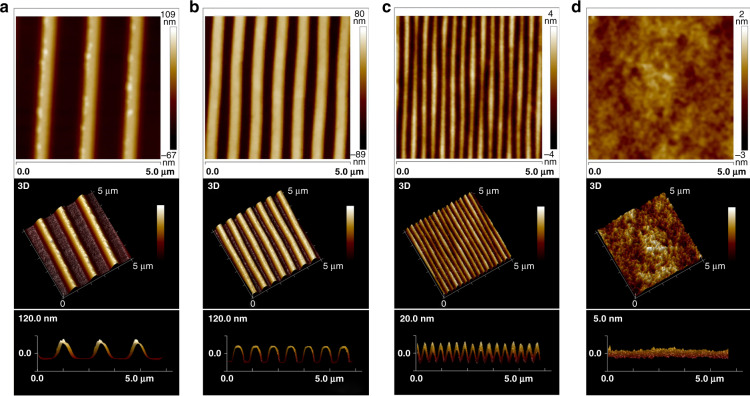
AFM images of PDMS-based nanotopographical and flat surfaces replicated from different optical media in 2D and 3D (top view and side view). **a** CD-R (groove width: 890 nm; ridge width: 520 nm; ridge height: 110 nm). **b** DVD-R (groove width: 420 nm; ridge width: 320 nm; ridge height: 110 nm). **c** BD-R (groove width: 190 nm; ridge width: 120 nm; ridge height: 17 nm). **d** Flat PDMS (average R_a_: 1.02 nm)

The PDMS substrates with nanoscale surface patterns transferred from the optical discs, including the CD-R, DVD-R, and BD-R discs and flat PDMS substrates were also measured using AFM, as shown in Fig. 3. The widths of the grooves and ridges on these PDMS-based surfaces were approximately 890 and 520, 420 and 320, and 190 and 120 nm, respectively. Both depths of the nanogrooves duplicated from CD-R and DVD-R were close to 110 nm, while the depth was approximately 17 nm for the BD-R replica. Thus, the nanogrooved surface patterns exhibited high fidelity with dimensional tolerances below 20% during rapid PDMS nanomolding, as demonstrated in Fig. S2. In addition, Fig. S3a, b sequentially presents the magnified nanogrooves on the BD-R surface and on its replicated PDMS surface via AFM before and after oxygen plasma etching, indicating that the nanotopographies remained good after the surface treatment.

Moreover, in the present study, the Young’s modulus (*E*_*r*_) of different PC/PDMS substrates was measured because material stiffness also plays an important role in the regulation of cell behaviors. As shown in Fig. S3c, PC substrates obtained from different optical discs had similar stiffnesses, and their average value of *E*_*r*_ was 2.23 GPa, while it was 2.75 MPa for PDMS. In addition, the measurement of surface roughness (*R*_*a*_) for various PC/PDMS substrates was performed, as shown in Fig. S3d, since roughness affects the interactions between the cells and their contact surfaces, including cellular adhesion and protein adsorption. From the results, both *R*_*a*_ values of flat PC and flat PDMS were a few nanometers, both *R*_*a*_ values of BD-R and its PDMS replica were also less than 10 nm, and the *R*_*a*_ values of CD-R, DVD-R, and their PDMS duplications increased slightly by 30–40 nm.

### Muscle cell growth on nanogrooved/flat PC/PDMS

**Fig. 4:**
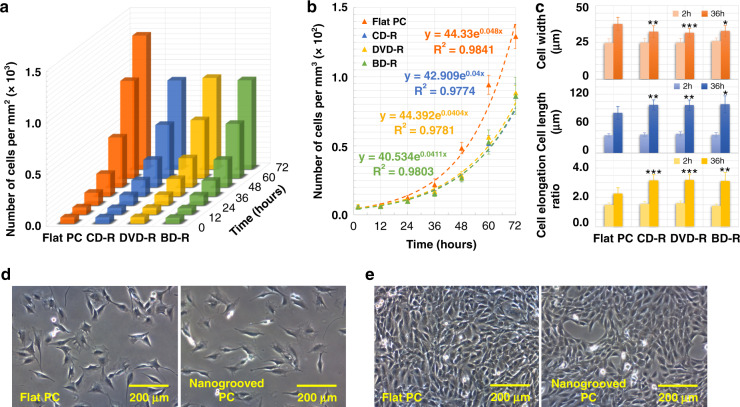
Muscle cell growth on different PC substrates. **a** 3D chart of C2C12 cell proliferation rates within 3 days on the nanogrooved PC surfaces from different optical media, including the CD-R, DVD-R, and BD-R discs, and the flat PC substrate. **b** 2D diagram of curve fitting for the cell growth rates in **a**. Error bars represent the SD (n > 50,000 cells). **c** Average width, length, and elongation of myoblasts that grew on the corresponding PC substrates at 2 and 36 h after cell seeding. Error bars represent the SD (*n* = 400 cells). *P < 0.05, **P < 0.01, ***P < 0.001, compared to the flat PC substrate. Data are results from 3 independent experiments. Optical microscopic images of cell growth on flat and nanogrooved (groove width: 100 nm; ridge width: 200 nm; ridge height: 20 nm) PC substrates at different times after cell seeding: **d** 1 day, **e** 3 days

C2C12 myoblasts were seeded on each piece of PC substrate (2 × 2 cm) prepared from different optical discs at the same density of 1 × 10^4^ cells/cm^2^, and they were cultured on flat PC as a control group. Microscopic observation showed that the muscle cells in growth medium (GM) attached, elongated, migrated, and proliferated for 3 days (Fig. 4d) until completely covering the surfaces (Fig. 4e). More cells on the flat surface were observed than on other nanogrooved substrates with the same incubation time, as demonstrated in Fig. S4. During this period, the number of cells per square millimeter and the cell morphology, such as width and length, were recorded to calculate the cell growth rate and the cell elongation, respectively. Figure 4a shows the speed of myoblast proliferation on various PC surfaces. The orange column represents the number of cells cultured on the flat substrate within the next 3 days after cell seeding. The blue, yellow, and green columns indicate the numbers of cells cultured on the nanogrooved surfaces of the CD-R, DVD-R, and BD-R discs, respectively. The results revealed that the myoblasts proliferated the fastest on the flat surface, whereas all the C2C12 cells induced by various nanotopographies grew slowly, as the height of the column reflects the rate of muscle cell growth. Furthermore, the speed of myoblast proliferation can be quantified using

$$N_t = N_0 \ast e^{gr \ast t},$$

where *N*_*t*_ is the cell number at time *t*, *N*_*0*_ is the initial number of cells, *t* is the culture time, and *gr* is the cell growth rate. The calculation via the curve-fitting technique revealed that the estimated *gr* values were 0.040, 0.040, 0.041, and 0.048 per hour for the muscle cells on the CD-R, DVD-R, BD-R, and flat PC surfaces, respectively. As shown in Fig. 4b, 10 nonoverlapping areas were randomly selected with over 50,000 cells counted; the error bars represent the standard deviation (SD) of each dataset. Correspondingly, the cell doubling time (*T*), which is defined as *T* = *1/gr*, was equal to 25.00, 24.75, 24.39, and 20.83 h. The shorter the time required for doubling the number of myoblasts is, the faster the cell proliferation speed. Therefore, the qualitative and quantitative results were in agreement that the nanotopographical cues on PC substrates remarkably reduced the muscle cell growth rate by approximately 19% on average, and no obvious difference was found between the groove patterns with different dimensions at the nanoscale.

Moreover, the myoblasts attached to the nanogrooved PC surfaces appeared markedly stretched compared with the cells on the flat substrate, which showed an elliptical shape. Thus, the effect of nanotopography on cell morphology was further quantified by calculating cell elongation. The widths and lengths of the C2C12 cells cultured on different PC surfaces were measured using ImageJ software after cell seeding for 2 and 36 h to obtain the cell elongation values, which are defined as *length/width*. As shown in Fig. 4c, the values of cell elongation for the myoblasts cultured on the CD-R, DVD-R, BD-R, and flat PC surfaces for 2 h were 1.55, 1.60, 1.42, and 1.49, respectively; after 36 h, they became 3.16, 3.19, 3.12, and 2.26, respectively. Each data point represents the mean value of 5 randomly selected field-of-views (FOVs), and 400 cells were counted. This finding indicated that the muscle cells guided by nanotopography were obviously elongated by an average of approximately 40%.

**Fig. 5:**
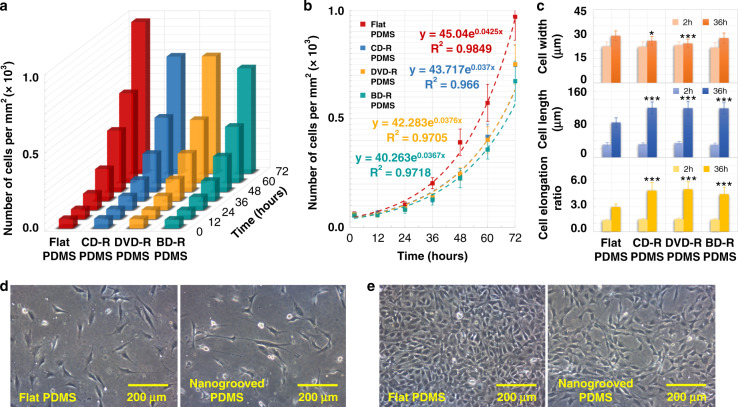
Muscle cell growth on different PDMS substrates. **a** 3D chart of C2C12 cell proliferation rates within 3 days on the nanogrooved PDMS surfaces replicated from different optical media, including the CD-R, DVD-R, and BD-R discs, and the flat PDMS substrate. **b** 2D diagram of curve fitting for the cell growth rates in **a**. Error bars represent the SD (*n* > 50,000 cells). **c** Average width, length, and elongation of myoblasts that grew on the corresponding PDMS substrates at 2 and 36 h after cell seeding. Error bars represent the SD (*n* = 400 cells). *P < 0.05, ***P < 0.001, compared to the flat PDMS substrate. Data are results from 3 independent experiments. Optical microscopic images of cell growth on flat and nanogrooved (groove width: 190 nm; ridge width: 120 nm; ridge height: 17 nm) PDMS substrates at different times after cell seeding: **d** 1 day, **e** 3 days

The PDMS substrate with a nanogrooved surface provided not only nanotopographical cues but also low stiffness for myoblasts in vitro, which are both close to the cellular microenvironment in vivo. Thus, the growth of muscle cells on these functional PDMSs is worth investigating. C2C12 myoblasts were seeded on the patterned and flat PDMS surfaces at the same cell density of 1 × 10^4^ cells/cm^2^. Similar to the state of cell growth on PC substrates, myoblasts adhered, spread (Fig. 5d), and proliferated until they fully covered the PDMS surfaces (Fig. 5e), and more cells were also observed on smooth PDMS (Fig. S5). The number of cells cultured on the PDMS substrates with different surface topographies was counted over the first 3 days after cell seeding, as illustrated in Fig. 5a. The results demonstrated that the cells grew on flat PDMS more rapidly than on other nanogrooved surfaces, as evidenced by the corresponding red column being the highest. Based on the calculation shown in Fig. 5b, the *gr* and *T* values were 0.037 and 27.03, 0.038 and 26.60, 0.037 and 27.25, and 0.043 and 23.53 for the myoblasts proliferated on the PDMS substrates with surface features molded from the CD-R, DVD-R, BD-R, and flat PC surfaces, respectively. Each data point was expressed as the average of 10 randomly selected areas, and over 50,000 cells were counted in total. Thus, the muscle cells on the PDMS with various nanotopographies grew, on average, approximately 15% slower than the myoblasts on the flat PDMS surface, while the effect of the size difference of the groove patterns at the nanoscale on cell proliferation was not significantly different. In addition, the C2C12 myoblasts attached to the nanopatterned PDMS exhibited prominently elongated shapes. Figure 5c also reveals cell elongation values of 1.48, 1.52, 1.46, and 1.38 at 2 h and 4.98, 5.14, 4.54, and 2.81 at 36 h in the cases of different PDMS substrates with surface features molded from the CD-R, DVD-R, BD-R, and flat PC surfaces, respectively. Each data point represents the mean value of 5 randomly selected FOVs, and 400 cells were counted. Therefore, the cell body on PDMS surfaces was linearly lengthened by an average of approximately 74% as a result of the nanotopographical cue.

Comparing the experimental results of cell culture on various PC and PDMS substrates together, as shown in Fig. S6a, C2C12 cells grew fastest on the flat PC surface, while myoblasts proliferated slowly the most on the nanogrooved PDMS. Additionally, the myoblasts on the PDMS with nanotopographies appeared elongated with the highest elongation rate, while the morphology of the cells on the smooth PC was closest to a circle, as shown in Fig. S6b. It was calculated that the proliferation rate of C2C12 cells on flat PDMS decreased by approximately 13% and that the cell shape was linearly elongated by approximately 24% compared with myoblasts on flat PC. For the cells on nanogrooved PDMS, these two values further increased to approximately 29% and 116% on average. Thus, both nanotopographical cues and substrate stiffness regulated cell growth behavior, including the proliferation rate and morphology of C2C12 myoblasts. Specifically, nanoscale groove patterns and soft polymer materials were capable of slowing down the myoblast proliferation speed and inducing cell elongation independently as well as synergistically, and the effects could be added together when influenced by both of these biophysical cues simultaneously. In addition, the average growth rate of cells on nanogrooved PC was lower than the speed of cell proliferation on flat PDMS; correspondingly, the cell elongation caused by nanotopography was more pronounced than that due to low material stiffness, indicating that nanotopographical cues had a greater effect. Moreover, the different groove dimensions, ranging from tens of nanometers to hundreds of nanometers, exhibited similar influences on myoblast proliferation and elongation. In particular, the nanogrooves on the BD-R format, with a width of 100 nm and a depth of 20 nm, which were smaller than the previously reported critical dimensions that C2C12 cells were perceived to align, had been proven to affect muscle cell growth as well. In addition, it is worth noting that the direction of cell elongation for the cells on the PDMS with DVD-R patterns was nearly consistent with the direction of the underlying nanogrooves within 1–2 days after cells were seeded, whereas this phenomenon was not obvious in other cases.

### Myotube differentiation on nanogrooved/flat PC/PDMS

**Fig. 6:**
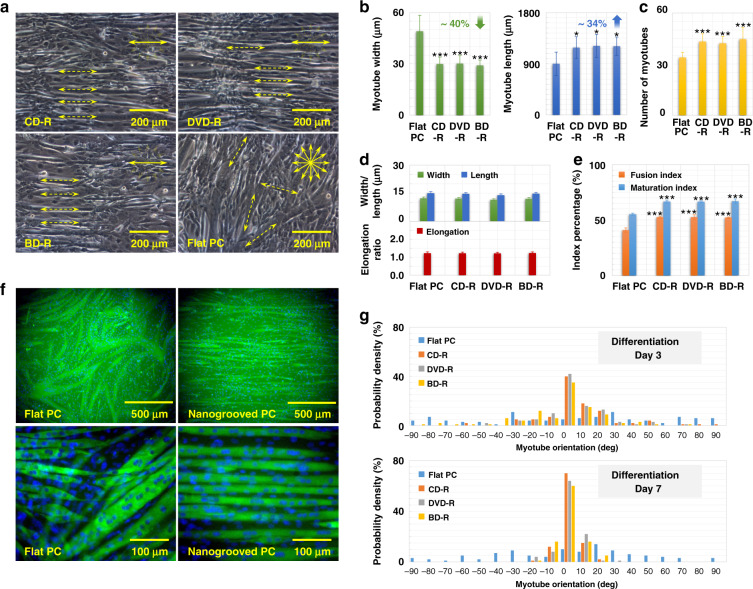
Muscle cell differentiation on different PC substrates. **a** Optical microscopic images of differentiated C2C12 myoblasts at the 10th day after cell seeding on the CD-R, DVD-R, BD-R, and flat PC surfaces. The directions of the nanogrooved patterns are marked by solid arrows, while the directions of the myotubes on the surfaces are marked by dotted arrows. **b** Average width and length of myotubes that grew on the corresponding PC substrates. Error bars represent the SD (*n* = 400 myotubes). **c** Average number of myotubes cultured on the corresponding PC substrates. Error bars represent the SD (*n* = 1638 myotubes). **d** Average width, length, and elongation of the cell nucleus in myotubes that grew on the corresponding PC substrates. Error bars represent the SD (*n* = 400 nuclei). **e** Fusion index and maturation index for myoblasts differentiated on the corresponding PC substrates. Error bars represent the SD (*n* = 971 myotubes). *P < 0.05, ***P < 0.001, compared to the flat PC substrate. Data are results from 3 independent experiments. **f** Fluorescence images of myotubes differentiated for 5 days on flat and nanogrooved (groove width: 100 nm; ridge width: 200 nm; ridge height: 20 nm) PC surfaces. Myosin was stained green, while the cell nucleus was stained blue. **g** Orientations of myotubes that initiated their differentiation on the nanogrooved/flat PC substrates for 3 and 7 days

C2C12 myoblasts that overgrew on PC substrates were continuously cultured in culture medium for another week. The GM was replaced with differentiation medium (DM) to initiate cell differentiation. During this process, the closely arranged and mononucleated myoblasts fused with one another, giving rise to small and nascent myotubes with few nuclei. Subsequently, the surrounding myoblasts continued to fuse with the nascent myotubes, finally forming mature multinucleated myotubes over the entire surfaces. Figure 6a demonstrates the differentiated myoblasts on the 10th day after cell seeding on the CD-R, DVD-R, BD-R, and flat PC surfaces. Through observation, all C2C12 cells cultured on various PC substrates could differentiate into myotubes, while most myotubes on the nanotopographical surfaces were long, thin, and linear in shape and headed in the same directions as their underlying nanogrooves (marked by dotted arrows) without distinct differences caused by the different groove sizes. Meanwhile, on the smooth PC surface, the myotubes appeared short, wide, partly branched, and grew in a random fashion (marked by dotted arrows).

Furthermore, the length and width of those myotubes were measured to analyze and quantify the effect of nanotopography on myotube morphology. As shown in Fig. 6b, the myotubes that differentiated on the nanogrooved PC substrates were more than 1100 µm in length and approximately 30 µm in width on average, while those cultured on the smooth surfaces were less than 900 µm in length and approximately 50 µm in width. Each data point represents the average value of 6 randomly selected FOVs with 100 myotubes counted. Thus, the differentiated myotubes elongated by approximately 34% and narrowed by approximately 40% under the guidance of nanotopographical cues. The orientation of the myotubes, which is defined as the angle between a myotube and its bottom nanogrooves, was recorded to assess the cell alignment under the nanotopographical cue. The orientation angle was treated as 0° when a myotube was aligned parallel to the underlying grooves. Moreover, the angle of ±90° indicated that a myotube was aligned perpendicular to the surface patterns. As shown in Fig. 6g, the orientations of the myotubes on the optical discs tended to be distributed in the range of ±10°, and this trend became more obvious over time. The orientation angles for the myotubes differentiated on the flat PC were almost uniformly distributed between ±90°. Thus, the myotubes were guided by the nanotopographical cue to become longer and thinner and align with the nanogroove direction. Otherwise, in the absence of surface nanotopography, the myotubes maintained the shapes of original differentiation with random directions.

Immunofluorescence cell staining was applied to quantify cell maturation, which further revealed the influence of nanotopography on skeletal muscle cell differentiation. As demonstrated in Fig. 6f, myotubes differentiated for 5 days on nanogrooved and flat PC surfaces were fixed and stained with fluorescent dyes, including anti-myosin heavy chain Alexa Fluor 488 (eBioscience) and DAPI (Invitrogen). Myosin, which only appears in mature myotubes and plays a critical role in cell contraction, was stained green for the recognition of myotubes, while the cell nucleus, which is present in every cell body, was stained blue for the assessment of cell maturity. The results of fluorescence staining revealed the number of myotubes and cell nuclei counted for the calculation of the fusion index and the maturation index, as shown in Fig. 6c, e. Each data point represents the mean value of 10 randomly selected areas; approximately 2000 myotubes and over 10,000 nuclei were counted in total. The fusion index refers to the myotube nucleus number divided by the total number of cell nuclei, the maturation index is equal to the number of myotubes with more than five nuclei divided by the total number of myotubes, and both indices are indicators of myotube maturation. The results showed that the fusion and maturation indices were 0.53 and 0.67, 0.53 and 0.66, and 0.52 and 0.67 for the cells cultured on the nanogrooved surfaces of CD-R, DVD-R, and BD-R, respectively, while on the flat PC surfaces, the two indices were 0.41 and 0.55, respectively. Therefore, the two indices of maturity under nanotopographical induction were greater than the corresponding values of the cells differentiated on flat PC substrates by approximately 28 and 21% on average, indicating that the differentiation of skeletal muscle cells was significantly improved with enhanced maturation under the guidance of nanotopographical cues. In addition, as shown in Fig. 6d, no obvious elongation of the nucleus was found within the myotubes on the PC substrates.

**Fig. 7:**
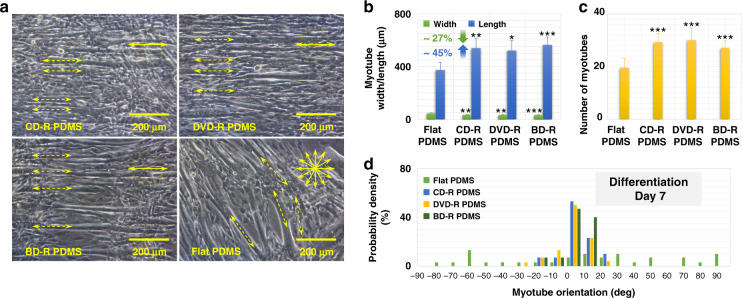
Muscle cell differentiation on different PDMS substrates. **a** Optical microscopic images of differentiated C2C12 myoblasts on the 10th day after cell seeding on PDMS surfaces duplicated from CD-R, DVD-R, BD-R, and flat PC. The directions of the nanogrooved surface are marked by solid arrows, while the directions of the myotubes on the surfaces are marked by dotted arrows. **b** Average width and length of myotubes that grew on the corresponding PDMS substrates. Error bars represent the SD (*n* = 240 myotubes). **c** Average number of myotubes cultured on the corresponding PDMS substrates. Error bars represent the SD (*n* = 1055 myotubes). *P < 0.05, **P < 0.01 ***P < 0.001, compared to the flat PDMS substrate. Data are results from 3 independent experiments. **d** Orientations of myotubes that initiated their differentiation on the nanogrooved/flat PDMS substrates for 7 days

Similar to the DM culture of the muscle cells that grew all over the surfaces of different optical discs for further differentiation, the C2C12 myoblasts on the PDMS substrates were also continuously cultured for another week after they completely covered the surfaces. Cell differentiation was initiated and maintained in GM instead of DM because the differentiated myotubes easily detached from the PDMS surfaces in the DM. As shown in Fig. 7a, on the 7th day after the initiation of cell differentiation, the myoblasts on the patterned and smooth PDMS substrates replicated from the CD-R, DVD-R, BD-R, and flat PC surfaces all differentiated into myotubes. Figure 7b, c also shows the measurement results of the average width, length, and number of myotubes, which were approximately 370 µm in length and 40 µm in width for the cells cultured on the flat PDMS surface. In the case of the PDMS-based nanotopographical surfaces, the average myotube length was approximately 540 µm, and the width was approximately 30 µm, indicating a 45% increase in length and a 27% reduction in width. Data in Fig. 7b were from 6 randomly selected FOVs with 240 myotubes analyzed, and data in Fig. 7c were from 10 randomly selected areas with 1055 myotubes analyzed. Furthermore, myotube alignment was evaluated by measuring the cell orientation, as demonstrated in Fig. 7d. The orientation angles between the myotubes on the PDMS with nanotopographies and their underlying nanogrooves were largely within the range of ±10°, and a few fell outside the range of ±20°, indicating parallelly aligned myotubes. In contrast, the orientations of the myotubes on the PDMS without any surface pattern were almost uniformly distributed over the entire range, indicating myotubes with arbitrary directions.

From the results, during myogenesis, C2C12 myoblasts actively responded to the nanotopographical cue from the grooved surfaces by differentiating into parallelly aligned myotubes with elongated morphology and enhanced maturation, regardless of substrate stiffness. However, in the presence of low material stiffness alone, no cell alignment was observed. In addition, different dimensions of groove patterns in the nanometer range showed no distinct difference in skeletal muscle cell differentiation.

### Skeletal muscle contraction

**Fig. 8:**
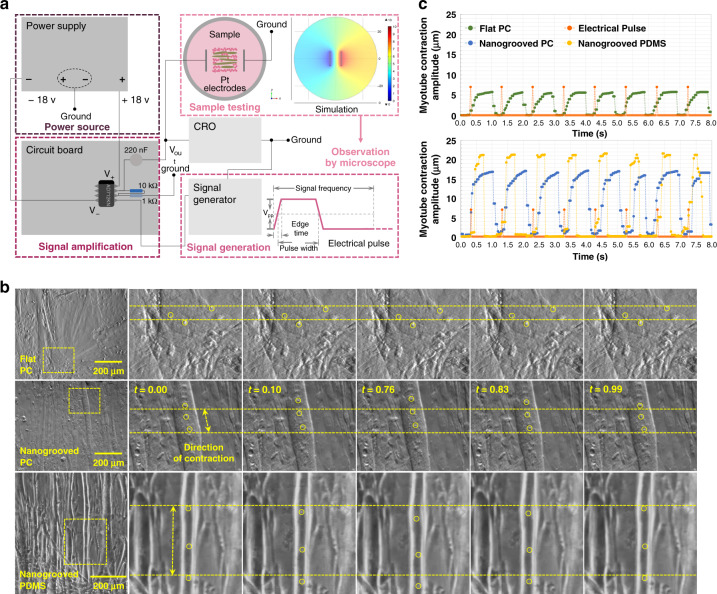
Muscle cell contraction under electrical stimulation. **a** Schematic of the electrical stimulation system, which consisted of three parts: signal generation, signal amplification, and sample testing area. **b** Sequences of optical microscopic images taken from videos of cell contraction on flat and nanogrooved (groove width: 100 nm; ridge width: 200 nm; ridge height: 20 nm) PC surfaces and nanogrooved (groove width: 190 nm; ridge width: 120 nm; ridge height: 17 nm) PDMS substrates. **c** Displacements of the contractile myotubes cultured on the corresponding nanogrooved/flat PC and PDMS surfaces of **b** under electrical stimulation

Cell contraction, which plays an important role in muscular movement, is the main function of mature myotubes. As shown in Fig. 8a and Fig. S7a, an electrical stimulation system was developed to provide myotubes cultured in vitro on the platform with continuous electric pulses. Under the stimulation of electrical pulses, the well-differentiated myotubes contracted on all the nanogrooved and flat PC and PDMS substrates from the optical media, and the contractile activity was recorded by a video camera under an optical microscope. The motions of small regions within myotubes during one pulse were captured from a time lapse series of images. Then, the contraction frequency and amplitude were tracked frame by frame and quantified as a function of time by ImageJ software, as shown in Fig. 8c. The sequential images in Fig. 8b demonstrate the changes in the position of the representative areas within the myotubes cultured on nanogrooved/flat PC and PDMS substrates under a single electrical pulse. To investigate the effects of nanogrooves on cell contraction, preliminary studies were performed to analyze the contraction displacements of 60 myotubes: 20 of them were cultured on flat PC substrates; 20 of them were cultured on nanogrooved PC substrates; and 20 of them were cultured on nanogrooved PDMS substrates. The results revealed that under the same conditions of electrical stimulation (pulses: *V*_*pp*_ = 7 V; *f* = 1 Hz; *t*_*pw*_ = 10 ms; *t*_*e*_ = 5 ns), the cell contraction frequency was consistent with the pulse frequency. As shown in Fig. S8, the myotubes on PC and PDMS substrates induced by nanotopography achieved average longitudinal contraction displacements of 14.59 µm and 19.32 µm, respectively, which were significantly larger than the average contraction distance of 5.32 µm observed in the cells on flat PC surfaces. (Note: The data for myotubes on flat PDMS substrates is not provided here because myotubes are easily detached from PDMS surfaces.) Additionally, the nanotopography-induced skeletal muscle cells contracted along the alignment direction of their underlying nanogrooves, whereas the contraction direction was random in the absence of nanotopographical cues. Moreover, since Ca^+^ plays an important role in muscle contraction, Fig. S7b demonstrates the changes in Ca^+^ fluorescence intensity within the myotubes cultured on patterned and flat PC surfaces during a stimulus. Correspondingly, the intensity profiles of the marked positions were plotted, as shown in Fig. S7c. From the results, myotubes on the grooved surface exhibited a more than 60% increase in fluorescence intensity, while the average value was 20% for the cells on the flat substrate. Thus, the contraction of myotubes was improved by the nanotopographical cue.

## Discussion

Multiple cues regulate cells in their natural microenvironment. Among these cues, the surface topography at the nano and microscales, which is independent of biochemistry, has cell-specific influences on cellular behavior through physical contact guidance^50,51^. The phenomenon of contact guidance, which refers to the effect of topographical patterns on the orientation of attached cells, was recognized decades ago^52,53^. Unlike the geometric constraint at the microscale^54^, the nanotopography in which the feature size is much less than the cell scale guides cell growth in a distinct manner through cell sensing, while the cellular response depends on the cell type. For instance, human corneal epithelial cells exhibited a lower growth rate on nano-gratings^55^, whereas human mesenchymal stem cells proliferated faster on aligned carbon nanotube networks^56^. Moreover, cells are extremely sensitive to the nanofeature size. As previously reported, nanotopography-induced alignment of human embryonic kidney cells and skeletal myoblasts was observed only above a critical dimension^32^.

In this work, skeletal muscle cells were cultured on PC and PDMS substrates (flat or with nanogrooves), with groove/ridge widths ranging from 100 to 900 nm and depths of approximately 20 to 120 nm. Compared to cells on flat substrate surfaces, the proliferation rates were found to be decreased by the nanotopographical cue by as much as 19 and 15% for myoblasts on PC and PDMS surfaces, respectively. Correspondingly, the cell elongation values were increased by as much as 40 and 74%, respectively. From these results, we can conclude that nanotopographical cues independently regulated the proliferation and elongation of cells, regardless of the substrate material. Moreover, a decrease in substrate material stiffness was observed to cause a reduction in the proliferation rate and elongation of the cell shape. Comparing cells cultured on *flat* PC (with a Young’s modulus of 2.23 GPa) and *flat* PDMS substrates (with a Young’s modulus of 2.75 MPa), the cell proliferation rate was decreased by 13%, and the cell shape was elongated by 24% for the cells on the softer surface (i.e., PDMS surface). Similarly, comparing the cells cultured on the PC and PDMS substrates with the *same nanogrooved patterns*, the values of the decreased proliferation rate and the increased elongation were 8 and 58%, 7 and 61%, 12 and 46% for the cells on the softer surfaces, i.e., PDMS surfaces molded with CD-R, DVD-R, and BD-R surface patterns, respectively. From these experimental observations, it is clear that the biophysical cue of substrate *stiffness* independently affects the proliferation and elongation of cells, regardless of the surface topography. Between the two influential factors, nanotopographical cues showed a higher effect.

Moreover, comparing the cells cultured on the flat and nanogrooved PC substrates as well as on the flat and nanogrooved PDMS substrates, cells grew *fastest* and in general with shapes similar to a circle on the flat PC surfaces, while cells proliferated *slowest* and in general grew in elongated shapes on the nanogrooved PDMS surfaces. We estimate that for the cells on the nanogrooved PDMS surfaces, their proliferation rate was reduced by 29% and their body was elongated by 116% compared with the cells on the flat PC surfaces. Based on these quantified results, the nanogrooved PDMS substrates provided two biophysical cues simultaneously, i.e., *lower stiffness* and *nanoscale surface features*, and had the greatest influence on cellular behaviors. The values of the decreased proliferation rate (i.e., 29%) and the increased elongation ratio (i.e., 116%) for the cells on nanogrooved PDMS surfaces were roughly close to the sum of the corresponding values for the cells on nanogrooved PC surfaces (i.e., 19 and 40%) and the cells on the flat PDMS surfaces (i.e., 13 & 24%). From the above data, we can conclude that the nanoscale structures and the soft polymer materials were capable of slowing the cell proliferation rate and inducing cell elongation synergistically, and their individual effects could be combined.

During myogenesis, mononucleated myoblasts differentiate into multinucleated myotubes through cell fusion, and mature myotubes are characterized by stimulated contraction. The fusion index, maturation index, and the number of myotubes on patterned substrates increased by 28, 21, and 30%, respectively, under the guidance of nanotopographical characteristics. On flat surfaces, the myotubes contracted under electrical stimulation at the same frequency of the given pulses, with an average amplitude of approximately 5.32 µm. In contrast, the myotubes cultured on nanogrooved PC and PDMS surfaces could achieve average amplitudes of 14.59 µm and 19.32 µm, respectively. In addition, orientation angles between myotubes and their bottom nanogrooves tended to be distributed in the range of ±10°, and this trend became more obvious over time. Therefore, the muscle cells actively responded to nanotopography by differentiation into parallelly aligned myotubes with enhanced maturation, regardless of the substrate stiffness. However, under the influence of multiple cues in the native ECM, it seems that cells grow at a slower speed in vivo than in vitro environments^14^. Through the investigation of nanotopography-induced muscle cells, myoblasts were also found to proliferate more slowly, and the differentiated myotubes became more mature. We assume that the reduced growth rate provides more time and opportunities for individual myoblasts to migrate, align with one another, and form long linear cell arrays, which further promotes cell differentiation because myoblasts tend to fuse in end-to-end configurations during myogenesis^57–59^. Furthermore, myotubes with enhanced maturation exhibit improved contraction function. More efforts will be made to explore their relationships and the underlying causes.

In addition, the difference between the muscle cells cultured on PC and PDMS substrates was assumed to be mainly caused by the difference in the material stiffness, which was 2.23 GPa and 2.75 MPa, respectively, in this study. However, many other factors, such as chemical composition, surface wettability, and functional groups, could also be different for the PC and PDMS materials, which may also affect protein adsorption, resulting in differences in cell growth. Therefore, further investigation should be performed to separate the effects of stiffness on cells from the effects of other factors. Further efforts will be made to distinguish the role of stiffness on cellular behaviors by using the same substrate materials with adjustable stiffness. Moreover, different feature sizes of the grooved patterns in the nanometer range showed mostly similar impacts on muscle cell growth and differentiation through the quantification and analysis of the related biological parameters. In particular, the shallow grooves with a 100 nm width and a 20 nm depth on the BD-R and its PDMS duplication also affected the cells. Therefore, this work sheds new light on the specific relationship between biophysical cues and muscle cell growth. Although myotube attachment on PDMS remains a problem that needs to be solved next, myoblast cultures using optical discs and their replicas enable parallelly aligned muscle cells on a large scale and have great potential to be applied in muscle regeneration and transplantation. In addition, the cell culture platform can be integrated with more ECM cues, such as various stiffnesses and proteins, for specific research purposes and cell types, further mimicking the native cellular microenvironment and contributing to the development of biomaterials in tissue engineering.

## Conclusions

In this study, we developed a novel strategy to aid the investigation of nanotopography-induced skeletal muscle cell growth and differentiation. Paralleled nanogrooves on commercially available optical discs were utilized to rapidly obtain flexible polymer substrates with nanoscale surface patterns on a large scale. The dimensions of the nanostructures on CD-R, DVD-R, and BD-R surfaces were characterized by AFM, ranging from tens to hundreds of nanometers. In particular, the dimensions of the grooves on the BD-R surface (i.e., 100 nm width by 20 nm depth) were smaller than the minimum pattern periodicity that myoblasts could sense for alignment, which was previously reported as 430 nm width by 100 nm depth. Moreover, PDMS platforms with low stiffness and various nanotopographies were generated through thermal nanomolding and surface treatment to further mimic the microenvironment of myoblasts in vivo and serve as functional substrates for guiding cells in vitro. By culturing C2C12 cells on nanogrooved/flat PC/PDMS surfaces and further quantifying the related biological parameters, myoblasts under the guidance of nanotopography and low stiffness showed a different growth pattern with a reduced proliferation rate and linearly elongated morphology and further differentiated into well-aligned myotubes with enhanced maturation and contractility.

## Materials and methods

### Preparation of optical discs

Commercially available optical discs including CD-R (700 MB, Maxwell, Hong Kong), DVD-R (4.7 GB, AccuCore, Sony, Hong Kong), and BD-R (25 GB, LTH Type, Verbatim, New York) were acquired for this study. Each disc was cut into 2 × 2 cm pieces, and the direction of concentric nanogrooves patterned on the PC layer was marked. Next, the protective layers of hard resin and the reflective layers of metal on top of the small disc pieces were peeled off with tweezers, leaving only the PC base layers attached to the recording layers of organic dye. Then, those disc pieces were treated with 30-min ultrasound cleaning of 75% ethanol and deionized water in sequence to completely remove the dye, and the resulting PC substrates with nanotopographical patterns were sterilized through 30-min UV exposure and stored in a sterile environment for future cell culturing. In addition, PC substrates with flat surfaces obtained from optical discs were also prepared following the same procedure. Prior to cell seeding, the PC substrates were treated with oxygen plasma (HARRICK Plasma Cleaner, HI RF Level, 750 mTorr) for 3 min to improve their hydrophilicity. In addition, atomic force microscopy (AFM, Bruker Dimension Icon) supported by NanoScope Analysis software (version 1.50, Bruker Corporation) and scanning electron microscopy (SEM, FEI Quanta 450 FEG) were used to characterize the nanogrooved surface patterns on the PC substrates.

### Fabrication of PDMS-based nanotopographical surfaces

Compared with the hard PC material, which has a Young’s modulus (*E*_*r*_) of 2–3 GPa, PDMS has a smaller stiffness by three orders of magnitude, which resembles the in vivo ECM around skeletal muscle cells more closely. Given that the biophysical cue of stiffness also plays an important role in regulating cellular behavior, the influence of PDMS-based nanotopography on muscle cells is a worthwhile endeavor. During the manufacturing process, soft lithography was applied to transfer the nanogrooved patterns obtained from the optical discs to the surfaces of PDMS substrates through rapid nanomolding. First, small pieces (2 × 2 cm) of PC substrates with nanogrooves and flat surfaces were prepared as templates, and PDMS (SYLGARD 184 Silicone Elastomer, Dow Corning) was mixed at a base-curing agent weight ratio of 10:1. Second, the liquid PDMS precursor was poured over the PC templates for full coverage, followed by degassing for 30 min and then postbaking at 70 °C for 2 h until fully cured. Third, the cured PDMS was peeled off, resulting in nanogrooved or smooth surfaces. Next, the PDMS substrates with/without nanogrooved surface patterns were sterilized under UV exposure and stored for future use. Moreover, treatment with oxygen plasma etching was required for the PDMS surfaces before cell culture experiments. AFM was also used to identify the nanotopographical features.

### Culture of skeletal muscle cells

The C2C12 cell line, an immortalized subclone of myoblasts, used in this study originated from satellite cells of thigh muscles of a 2-month-old C3H mouse. Mononucleated C2C12 myoblasts are generally capable of rapid proliferation under high serum conditions and further differentiation into multinucleated myotubes through cell fusion under low serum conditions. During myogenesis, certain genes are expressed to produce specific muscle proteins, which promote the formation of contractile skeletal muscles. For in vitro culture, C2C12 myoblasts were purchased from ATCC, maintained in growth medium (GM) containing Dulbecco’s modified Eagle’s medium (DMEM, ATCC) supplemented with 10% fetal bovine serum (Gibco) and 1% penicillin-streptomycin-neomycin (PSN, Gibco), and passaged when the cells reached 70–80% confluence. When the cells overgrew in culture dishes, GM was substituted with differentiation medium (DM) containing DMEM supplemented with 8% horse serum (Gibco) and 1% PSN to initiate myogenic differentiation and facilitate cell fusion. In particular, the mature myotubes were characterized by electrically stimulated contraction but little spontaneous contraction. In addition, a standard environment of 5% CO_2_ and 37 °C was provided by an incubator (Thermo Scientific Heracell 150i) for routine cell cultures, and the corresponding culture medium was renewed every 2–3 days.

In this study, C2C12 myoblasts were directly placed with a seeding density of 1 × 10^4^ cells/cm^2^ onto prepared PC/PDMS substrates with various nanotopographical patterns obtained from CD-R, DVD-R, and BD-R. The muscle cells were cultured in GM for 3 days, resulting in full confluence. The cellular morphology and the number of cells per square millimeter were recorded during this period to calculate the cell proliferation rates. The cells were then cultured in DM for another 1 week. Over this time, myoblasts differentiated into myotubes, as observed using an inverted microscope (Nikon ECLIPSE TS100), and immunofluorescence cell staining was applied to quantify the maturity of myotubes, as observed using a fluorescence microscope (Nikon ECLIPSE Ni). In addition, C2C12 cell culture on smooth PC/PDMS substrates without any patterns was also performed as the control.

### Electrical stimulation of muscle cell contraction

An electrical stimulation system was designed to provide continuous electric pulses to the in vitro cultured myotubes and investigate the cellular function of contraction. The system consisted of three components, including signal generation, signal amplification, and sample testing. Signal generation included a function/arbitrary waveform generator (Agilent 33250 A), a power supply (Manson DPD-3030), and an oscilloscope (Agilent MSO-X 2024 A), which generated electric pulses with adjustable signal frequency (*f*), voltage amplitude (*V*_*pp*_), pulse width (*t*_*pw*_), and edge time (*t*_*e*_). Furthermore, an amplifying circuit based on the OP Amp of AD712KN was developed to implement signal amplification, which modified the rising edge and the falling edge of the incoming impulses to avoid bubble generation in the culture medium. In the area of sample testing, a pair of platinum electrodes was placed in parallel on either side of the cell culture platform, with a constant spacing of 2 cm in between and 1 mm from the dish bottom, to give rise to an electric field. The COMSOL simulation demonstrated that the field distribution reached a maximum around the electrodes and gradually decreased with increasing distance from them. Before electrical stimulation, the cells were incubated in DM containing a 4.56 µM Fluo-4 AM calcium indicator (Invitrogen) for 1 h at 37 °C, washed in fresh DM and further incubated for 30 min. Subsequently, the input signals of electric pulses were generated and magnified in the system, stimulating the myotubes cultured on the PC/PDMS substrates via the electrodes. Meanwhile, the cellular contraction behavior and the resulting change in the fluorescence intensity of calcium ions (Ca^+^) were recorded by taking videos. Furthermore, video analysis and image processing were performed using ImageJ software to quantify the changes in frequency, amplitude, and intracellular Ca^+^ concentration of myotube contraction caused by electric stimuli.

### Statistical analysis

Each experiment for cell growth and differentiation was repeated independently 3 times with at least 3 samples. Each quantitative result for cell proliferation, elongation, and differentiation was expressed as the mean value of at least 5 randomly selected field-of-views (FOVs) with over 200 cells counted, and the error bars represent the SD for each dataset. For statistical analysis, Student’s two-tailed unpaired *t* test was performed, resulting in the differences between the cells cultured on nanogrooved and flat substrates. *P* < 0.05 is considered to be a significant difference.

## Supplementary information

Supporting Information

Supporting Information
