# Supplementary material for: Rapid nanomolding of nanotopography on flexible substrates to control muscle cell growth with enhanced maturation
Source: Microsyst Nanoeng. 2021 Nov 5;7:89. doi: 10.1038/s41378-021-00316-4 (PMC8571286; doi:10.1038/s41378-021-00316-4)
Supplement: Supplementary file 2 — Supporting Information [file 41378_2021_316_MOESM2_ESM.pdf]

Supporting Information

Rapid nano-molding of nanotopography on flexible substrates to control muscle cell growth with enhanced maturation

Cong Wu[a], Chriss S. M. Chin[a], Qingyun Huang[a], Ho-Yin Chan*[a], Xinge Yu[b], Vellaisamy A. L. Roy[c] and Wen J. Li[+a]

[a]Department of Mechanical Engineering, City University of Hong Kong, Hong Kong, China

[b]Department of Biomedical Engineering, City University of Hong Kong, Hong Kong, China

[c]James Watt School of Engineering, University of Glasgow, UK

*Corresponding author email: hoychan@cityu.edu.hk

[+] Corresponding author email: wenjli@cityu.edu.hk

Table of Contents

1. SEM characterization of nano-grooves on optical discs.

2. The dimensions of nano-grooves on discs and their PDMS replicas after nano-molding.

3. AFM characterization of nano-grooves on PC/PDMS surfaces.

4. Cell growth on nano-grooved/flat PC/PDMS.

5. Comparison of cell proliferation rate and morphology for cells on different surfaces.

6. Electrical stimulated cell contraction.

1. SEM characterization of nano-grooves on optical discs.

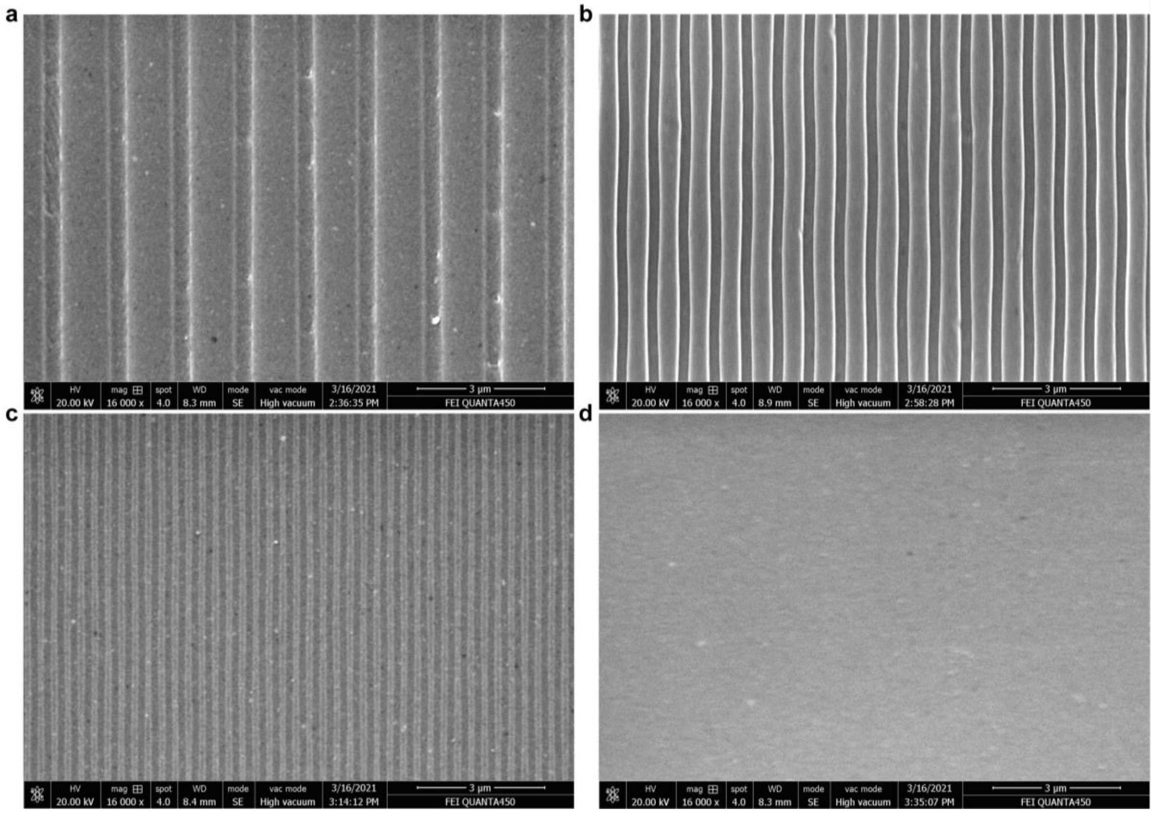

Figure S1. SEM images of nanotopographical and flat PC surfaces obtained from different optical media with a magnification of 16000 ×. (a) CD-R. (b) DVD-R. (c) BD-R. (d) Flat PC.

2. The dimensions of nano-grooves on discs and their PDMS replicas after nano-molding.

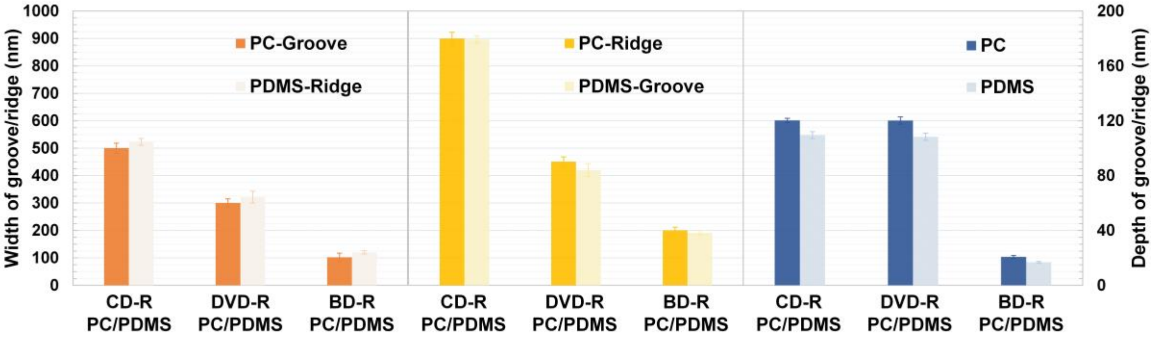

Figure S2. Average groove width, ridge width, and ridge height of the nano-grooved patterns obtained from the surfaces of CD-R, DVD-R, BD-R, and their corresponding PDMS replicas.

3. AFM characterization of PC/PDMS surfaces.

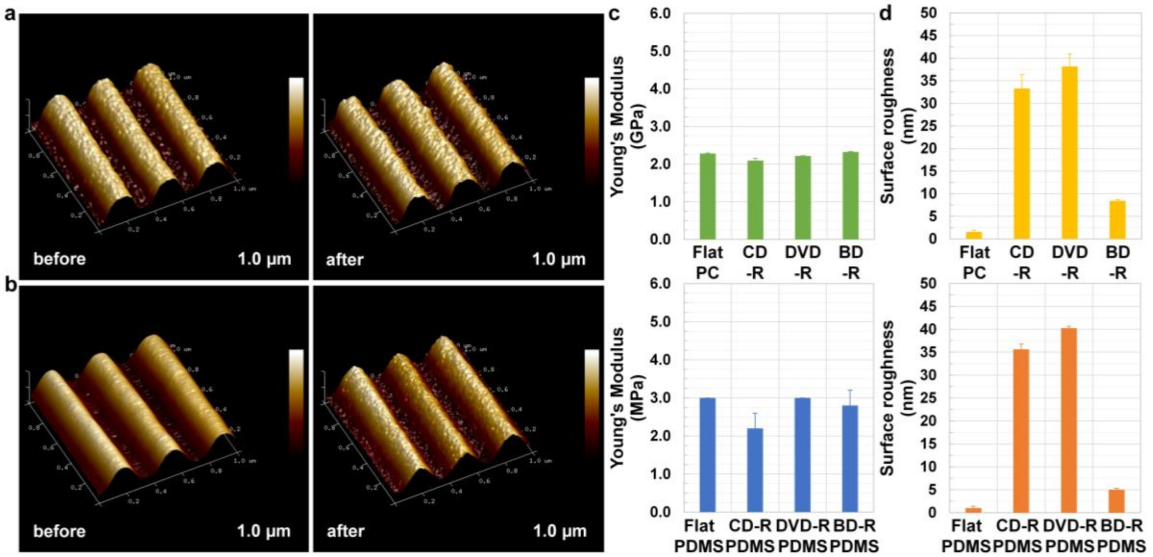

Figure S3. AFM images of the magnified nano-grooves with a scanning area of 1 µm × 1 µm on the BD-R surface (a) and on its replicated PDMS surface (b), before and after oxygen plasma treatment. Average Young's Modulus (c) and surface roughness (d) of the PC substrates obtained from CD-R, DVD-R, BD-R, and their corresponding PDMS duplications.

4. Cell growth on nano-grooved/flat PC/PDMS.

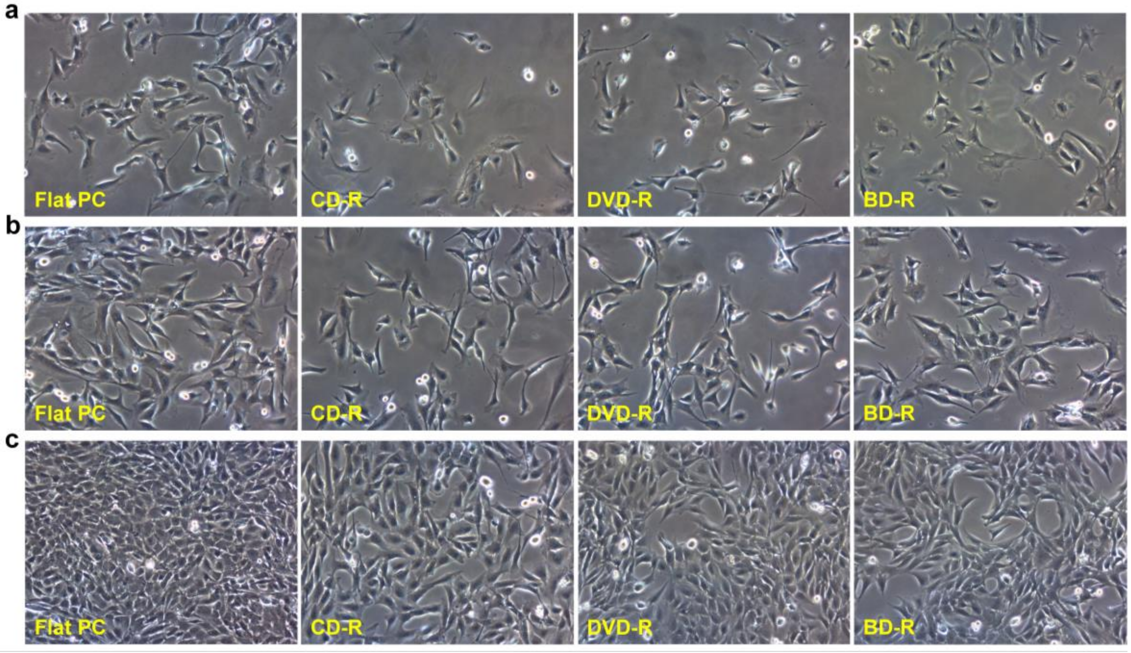

Figure S4. Optical microscopic images of cell growth on various PC substrates at different times after cell seeding: (a) 1 day. (b) 2 days. (c) 3 days.

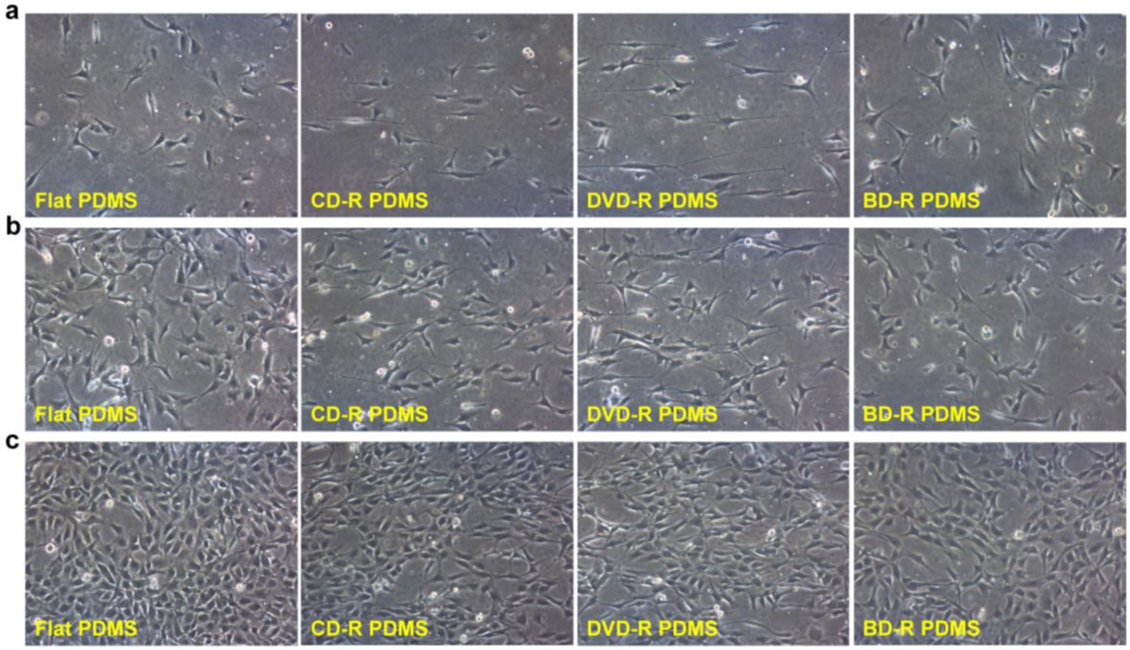

Figure S5. Optical microscopic images of cell growth on various PDMS substrates at different times after cell seeding: (a) 1 day. (b) 2 days. (c) 3 days.

5. Comparison of cell proliferation rate and morphology for cells on different surfaces.

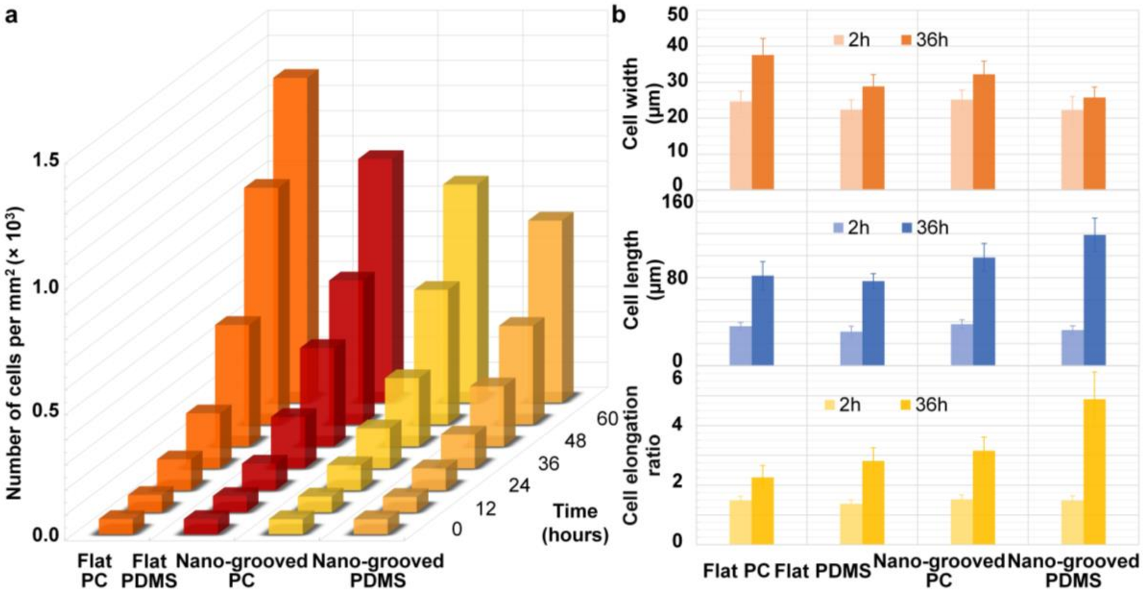

Figure S6. (a) 3D chart of average C2C12 cell proliferation rates within 3 days on the nano-grooved/flat PC/PDMS surfaces. (b) Average width, length, and elongation of myoblasts that grew on the nano-grooved/flat PC/PDMS surfaces at 2 and 36 h after cell seeding.

6. Electrical stimulated cell contraction.

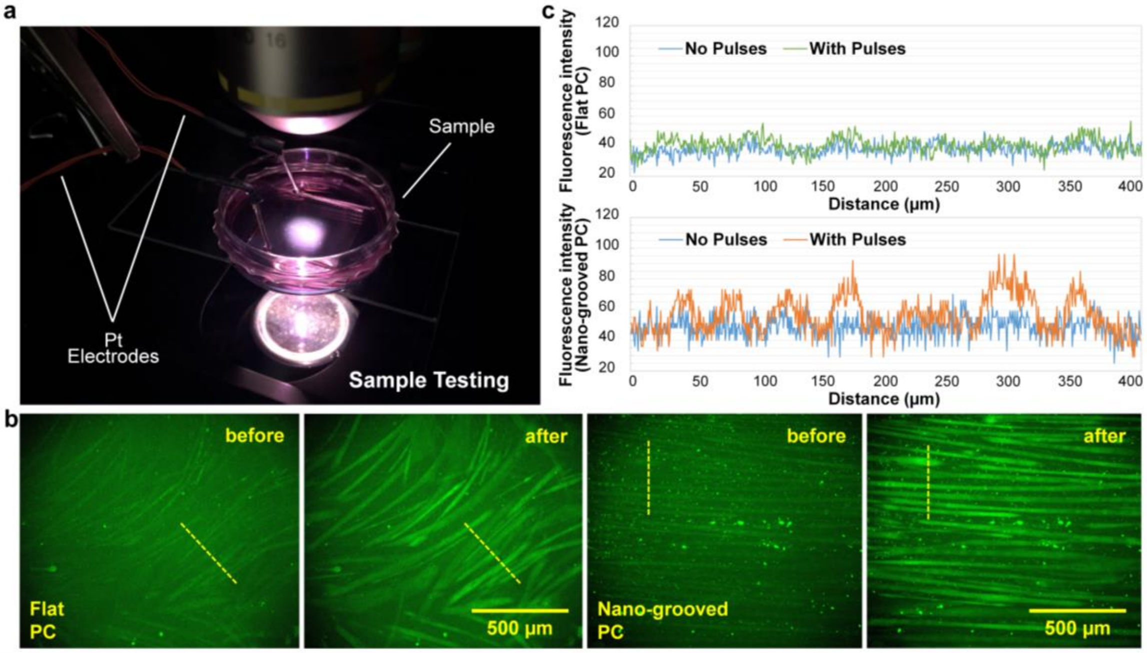

Figure S7. (a) Experimental setup of the sample testing area in the electrical stimulation system, including the cell culture platform, a pair of platinum electrodes for pulse output, and an optical microscope for real-time observation. (b) Fluorescence images of the myotubes labelled with Fluo-4 AM calcium indicator and cultured on the smooth and nano-grooved (groove width: 100 nm; ridge width: 200 nm; ridge height: 20 nm) PC substrates before and after an electrical stimulus. Ca^{+} was stained as green. (c) Fluorescence intensity of intracellular Ca^{+} along the corresponding profiles marked as dotted lines in (b).
